# Association between polymorphism of the *NEDD4* gene and cognitive dysfunction of schizophrenia patients in Chinese Han population

**DOI:** 10.1186/s12888-019-2386-y

**Published:** 2019-12-18

**Authors:** Chao Han, Kaiyan Cui, Xiaojiao Bi, Lina Wang, Mengmeng Sun, Limin Yang, Lanfen Liu

**Affiliations:** Shandong Mental Health Center, Jinan, Shandong People’s Republic of China

**Keywords:** *NEDD4*, Schizophrenia, Cognitive dysfunction

## Abstract

**Background:**

Schizophrenia is a complex psychiatric disorder with unknown etiology. A number of recent studies have shown that the polymorphism of the neural precursor cell expressed developmentally down-regulated 4 (*NEDD4*) gene is associated with a variety of neuropsychiatric disorders, such as schizophrenia, and may also be associated with cognitive dysfunction in these diseases.

**Methods:**

A case-control study was carried out, the alleles and genotypes distributions of five loci (rs3088077, rs2303579, rs7162435, rs11550869, rs62043855) of the *NEDD4* gene from 296 schizophrenia patients and 320 healthy controls were detected by using Taqman single-nucleotide polymorphism (SNP) genotyping technology. The clinical data of case and control group members were collected by self-made questionnaire and the psychotic symptoms of case group members were assessed by the Positive and Negative Syndrome Scale (PANSS). The Matrics Consensus Cognitive Battery (MCCB) was used to test the cognitive function of case group members.

**Results:**

The alleles and genotypes frequency of two loci (rs3088077, rs2303579) between case and control group showed significant differences (*P* <  0.05). There was no significant difference in MCCB scores of patients with different genotypes at rs3088077, rs11550869 and rs7162435 loci in case group. The study of rs2303579 locus showed that, patients’ scores with CT genotype were significantly lower than those with CC and TT genotypes (*P* <  0.05) in the test of Wechsler Memory Scale-Third Edition (WMS-III): Spatial Span, the scores of patients with TT genotype were significantly higher than those with CT genotype (*P* < 0.05) in the test of Hopkins Verbal Learning Test-Revised (HVLT-R). The study of rs62043855 locus showed that patients with TG genotype had significantly lower scores than those with GG genotype (*P* < 0.05) in the test of Neuropsychological Assessment Battery (NAB): Mazes.

**Conclusions:**

Our study showed that in schizophrenia patients of Chinese Han population, the polymorphisms of rs3088077 and rs2303579 loci were related to the pathogenesis of schizophrenia, while the polymorphisms of rs2303579 and rs62043855 loci were associated with cognitive dysfunction.

## Background

Schizophrenia is a common psychiatric disease with unknown etiology, which symptoms include cognitive dysfunction in addition to positive and negative symptoms [1]. Cognitive dysfunction, which may reflect genetic susceptibility, is now generally considered to be the core symptom of schizophrenia with its own pathological mechanisms and related to the abnormal development of the nervous system [2].

The ubiquitin-proteasome system (UPS), which participates in the intracellular signal transduction, synaptic function, plasticity regulation and neurodevelopment, is an important non-lysosomal protein degradation pathway in human cells [3, 4]. More and more studies show that UPS plays an important role in the pathological process of many neuropsychiatric diseases, including schizophrenia [5, 6]. The composition of UPS includes ubiquitin (Ub), ubiquitin-activating enzyme (E1), ubiquitin-binding enzyme (E2), ubiquitin ligase (E3) and 26S proteasome, among which E3 is the key factor in the selective degradation mechanism of UPS. The protein encoded by the neural precursor cell-expressed developmentally down-regulated 4 (*NEDD4*) gene belongs to the E3 family of ubiquitin ligase [7].

Current studies have shown that the *NEDD4* gene is associated with many neuropsychiatric disorders, such as schizophrenia [8]. Our previous studies have shown that multiple single-nucleotide polymorphisms (SNPs) of the *NEDD4* gene may be associated with schizophrenia, and the TT genotype of rs7162435 locus may be associated with the clinical phenotype of excitement and hostility in patients with schizophrenia [9]. The protein encoded by the *NEDD4* gene plays an important role in many physiological processes of the central nervous system. Most of these studies focus on degenerative diseases such as Huntington’s disease, Parkinson’s disease and Alzheimer’s disease [10, 11]. Current research on the *NEDD4* gene and cognitive dysfunction of schizophrenia is rare. Several studies have shown that abnormal expression of the NEDD4 protein in brain affects the ubiquitination of the α-amino-3-hydroxy-5-methyl-4-isoxazolepropionic acid (AMPA) receptor subunit glutamate receptor 1 (GluR1), and the dysfunction of GluR1 may be related to the pathogenesis of schizophrenia and its cognitive dysfunction [12–14].

Therefore, a case-control study was conducted by our research group to investigate the relationship between the *NEDD4* gene polymorphism and cognitive dysfunction in Chinese Han schizophrenia patients.

## Methods

### Subject recruitment

The study sample included 296 patients with schizophrenia and 320 healthy physical examinees (Table 1).

**Table 1:** The age and gender distributions of patients in case group and control group

Group	*N*	Age (χ ± s)	Gender	
			Male	Female
Case group	296	33.17 ± 10.99	108	188
Control group	320	32.48 ± 10.73	138	182
t/χ2		0.002	2.825	
*P*		0.430	0.093	

For the case group, all these patients were hospitalized in the Shandong Mental Health Center from March 2011 to December 2013, most of the patients who come to the hospital can cooperate to complete the psychological test, including MCCB test. The inclusion criteria were as follows: meeting the diagnostic criteria for schizophrenia in the Fourth Edition of the Diagnostic and Statistical Manual for Mental Disorders (DSM-IV); the diagnosis was made by at least two experienced psychiatrists according to the Structured Clinical Interview for DSM-IV (SCID); between the ages of 18 and 65 years old; biology parents were Chinese Han population; education level was junior high school and above; no anti-psychotic drugs were taken at least 1 month before entering the group (the time of inclusion was the day of hospitalization); no modified electric convulsive therapy was performed within 6 months before entering the group. The exclusion criteria were as follows: with other mental disorders except schizophrenia in DSM-IV; with major physical illness; in pregnancy or lactation.

For the control group, a total of 320 healthy physical examinees from March 2011 to December 2013 in Shandong Province were included. The inclusion criteria were as follows: between the ages of 18 and 65 years old; biology parents were Chinese Han population. The exclusion criteria were as follows: with schizophrenia or other mental illnesses in DSM-IV; with family history of mental illness; with major physical illness; in pregnancy or lactation.

### Scale selection

The general clinical data questionnaire made by our research team was used to collect clinical data of the participants, including their age, gender, education level, marital status, occupation and other disease-related data. The severity of the clinical symptoms of the case group members was assessed by using the Chinese version of Positive and Negative Symptom Scale (PANSS), which has been tested in China for norm and factor analysis and can be used to assess the symptoms of Chinese patients with schizophrenia [15]. We used the Chinese version of the Matrics Consensus Cognitive Battery (MCCB) to test the cognitive function of case group members. The reliability and validity of the Chinese version had been evaluated, which had been proven to be an effective tool for assessing cognitive deficits of Chinese patients with schizophrenia [16]. MCCB consists of the following ten subtests: Trail Making Test A (TMT-A); Brief Assessment of Cognition in Schizophrenia (BACS): Symbol Coding; Category fluency Test: Animal Naming (Fluency); Continuous Performance Test-Identical Pairs (CPT-IP); Letter-Number Span Test (LNS); Wechsler Memory Scale-Third Edition (WMS-III): Spatial Span; Hopkins Verbal Learning Test-Revised (HVLT-R); Brief Visuospatial Memory Test-Revised (BVMT-R); Neuropsychological Assessment Battery (NAB): Mazes; Mayer-Salovey-Caruso Emotional Intelligence Test (MSCEIT): Managing Emotions.

### SNPs selection

The SNPs information of the *NEDD4* gene in Chinese Han population was downloaded from the home page of the the International HapMap Project [17]. We analyzed these information by using Haploview 4.2 software and selected 9 Tagger SNPs with MAF > 0.05 and *r*^2^ ≥ 0.8, which were rs2303579, rs3088077, rs7162435, rs11550869, rs16976592, rs9806179, rs2414451, rs12593446 and rs7174459. Functional SNPs were obtained from the National Center for Biotechnology Information (NCBI) Database [18] and were searched according to the following principles: MAF > 0.05 in Chinese Han population or located in the 5’UTR region or the 3’UTR region or the exon. The functional SNPs we found were rs2303579, rs2302580, rs62043855, rs3088077, rs7162435, rs11550869, rs3833005, rs16976592, rs9920007, rs17238461 and rs1042477. Based on these information above, rs3088077, rs7162435, rs11550869 in the 3′ UTR region and rs2303579 (chr15:55860531, exon 4), rs62043855 (chr15:55915613, exon 1) in the exon were finally selected for research.

### DNA extraction and SNPs genotyping

Peripheral venous blood (5 mL) of control group and case group members was collect separately. The blood samples were shaken up in an anticoagulant tube containing 0.5 mol/L EDTA. The anticoagulant tubes containing the blood sample were centrifuged for 10 min at 3000 rpm/min to remove serum and white blood cells. The deoxyribonucleic acid (DNA) of the blood sample was extracted using the modified potassium iodide method. Genotyping was performed using the Taqman probe method of real-time quantitative polymerase chin reaction (Q-PCR). The primers and probes used in the experiment were shown in Table 2. The PCR reaction system was 10 μL, including 0.35 μL each of forward and reverse primers (20 pmol/μL), 0.35 μL each of FAM/HEX probes (10 pmol/μL), 5 μL of Premix, and 1.80 μL of double distilled water and 2 μL of DNA. The reaction conditions of PCR were as follows: denaturation at 95 °C for 3 min; reading at 95 °C for 15 s and 60 °C for 1 min, cycling 40 times; cooling at 4 °C for 30 min; 40 cycles in total.

**Table 2:** Primers and probes sequences of SNPs

SNP	Primer (5′-3′)	Probe (5′-3′)
rs3088077	Forward: GGCTGTGTTGCTTGATAGATGTTT	Probe 1: FAM-TTCCAGACCAcGAGCCCCTAGTG--TAMRA
	Reverse: GTCCCCAGCTGCAGACCTT	Probe 2: HEX-TTCCAGACCAtGAGCCCCTAGTGG-TAMRA
rs11550869	Forward: AAAGCACCTTCTGATTGTATAACACTTT	Probe 1: FAM-TCTGGAACTTCTGACAATCTGcCATGA-TAMRA
	Reverse: TACTTAACCTCTCTGGATTCATATTTCTTC	Probe 2: HEX-TCTGGAACTTCTGACAATCTGgCATGA-TAMRA
rs7162435	Forward: TACTGCTTTGTGGATCTTTAATGTTTG	Probe 1: FAM-ACCAATGGTCAAtAGGATATGCAGGCA-TAMRA
	Reverse: GCAATGGGTAAAAAGTATTAAAGCCT	Probe 2: HEX-TGGTCAAcAGGATATGCAGGCAAGA-TAMRA
rs2303579	Forward: TTACTTGACGGTGGAGGTGATG	Probe 1: FAM-AAGGCCTGGcTGC--MGB
	Reverse: TAAGAGAAGATGAAGCCACCATGTA	Probe 2: HEX-AAGGCCTGGtTGCT-MGB
rs62043855	Forward: CAGATGTCCTATGCATGAGCTTAATAT	Probe 1: FAM-CTGAATCAGAATtAAG-MGB
	Reverse: GTGATTGTAAACCAGAAATGTCAGAAA	Probe 2: HEX-CTGAATCAGAATgAAG-MGB

### Statistical analysis

In this study, SPSS 21.0 software was used to establish the database, and the goodness-of-fit Chi-square test was used to verify whether the allele and genotype frequencies of the members in the case group and control group corresponded to the Hardy-Weinberg equilibrium (HWE). The chi-square test was used to analyze the differences between the various qualitative data of the study subjects, including gender, family history, education level, allele frequency and genotype frequency, etc. Univariate analysis of variance, analysis of covariance and non-parametric tests were used to compare the differences in cognitive function of patients in the case group, and non-parametric tests were used for those with non-uniform variance. The LSD method was used to perform multiple analyses. All the analyses above were performed using SPSS 21.0 software. We performed a power calculation using the G*power program based on Cohen’s method [19]. All statistical tests were considered statistically significant at *P* < 0.05.

## Results

Data from 296 patients with schizophrenia and 320 healthy controls were analyzed. The results showed that there was no statistical difference in gender (χ^2^ = 2.825, *P =* 0.093) and age (t = 0.002, *P =* 0.430) between the two groups (Table 1). The present sample size revealed 99.86% power of detecting a significant association (α < 0.05) given an effect size index of 0.2 (corresponding to a weak to moderate gene effect).

### Hardy-Weinberg equilibrium

The genotype frequency distributions of the five SNPs in the control group all met HWE. In the case group, the genotype frequency distributions of four SNPs were consistent with HWE, except for rs11550869 (χ^2^ = 4.247, *P* = 0.039), the reasons may be as follows: firstly, the rs11550869 locus may be associated with the occurrence of disease or linked to disease susceptibility genes; secondly, the sample size is not large enough, and it may be necessary to increase the sample size to further clarify this issue.

### Comparison of allele and genotype frequencies between case group and control group

The allele frequencies of rs3088077 (χ^2^ = 15.4464, *P* < 0.001) and rs2303579 (χ^2^ = 8.301, *P* = 0.004) loci were significantly different between the case group and the control group, while the allele frequencies of rs11550869 (χ^2^ = 1.243, *P* = 0.265), rs7162435 (χ^2^ = 1.706, *P* = 0.192) and rs62043855 (χ^2^ = 1.080, *P* = 0.299) loci were not significantly different (Table 3).

**Table 3:** Comparison of allele frequencies of five SNPs in the *NEDD4* gene between case group and control group

SNP	Allele	Case group	Control group	χ^2^/*P*
rs3088077	C	354 (0.598)	451 (0.705)	15.464/< 0.001*
	T	238 (0.402)	189 (0.295)	
rs2303579	C	324 (0.547)	402 (0.628)	8.301/0.004*
	T	268 (0.453)	238 (0.372)	
rs11550869	G	93 (0.157)	89 (0.139)	1.243/0.265
	C	499 (0.843)	571 (0.861)	
rs7162435	T	413 (0.678)	468 (0.731)	1.706/0.192
	C	179 (0.302)	172 (0.269)	
rs62043855	T	383 (0.647)	432 (0.675)	1.080/0.299
	G	209 (0.353)	208 (0.325)	

Notes: * *P* < 0.05

The genotype frequencies of rs3088077 (χ^2^ = 14.961, *P* = 0.001) and rs2303579 (χ^2^ = 8.106, *P* = 0.017) loci were significantly different between the case group and the control group, while the genotype frequencies of rs11550869 (χ^2^ = 0.730, *P* = 0.694), rs7162435 (χ^2^ = 1.752, *P* = 0.416) and rs62043855 (χ^2^ = 1.047, *P* = 0.593) loci were not significantly different (Table 4).

**Table 4:** Comparison of genotype frequencies of five SNPs in the *NEDD4* gene between case group and control group

SNP	Genotype	Case group	Control group	χ^2^/*P*
rs3088077	CC	108 (0.365)	164 (0.513)	14.961/0.001*
	TT	50 (0.169)	33 (0.103)	
	CT	138 (0.466)	123 (0.384)	
rs2303579	CC	95 (0.321)	129 (0.403)	8.106/0.017*
	TT	67 (0.226)	47 (0.147)	
	CT	134 (0.453)	144 (0.450)	
rs11550869	GG	12 (0.041)	10 (0.031)	0.730/0.694
	CC	215 (0.726)	241 (0.753)	
	GC	69 (0.233)	69 (0.216)	
rs7162435	TT	143 (0.483)	171 (0.534)	1.752/0.416
	CC	26 (0.088)	23 (0.072)	
	TC	127 (0.429)	126 (0.394)	
rs62043855	TT	126 (0.426)	148 (0.463)	1.047/0.593
	GG	39 (0.132)	36 (0.112)	
	TG	131 (0.442)	136 (0.425)	

Notes: * *P* < 0.05

### Comparison of general clinical data and PANSS scores

We compared the differences of the general clinical data (age, gender, family history, smoking history, drinking history, history of psychosis, total disease duration) and the PANSS scores (PANSS scale total score, PANSS positive scale score, PANSS negative scale score, and PANSS psychopathology scale score) between different genotypes of each SNP. The results showed that the PANSS psychopathological scores of patients with different genotypes at rs2303579 locus were significantly different (*P* < 0.05). The ages of patients with different genotypes of rs11550869, rs7162435, and rs62043855 loci were significantly different (*P* < 0.05), and there was no significant difference in other general clinical data and PANSS scale scores between different genotypes of each five SNPs (*P* > 0.05).

### Comparison of cognitive functions

We used one-way analysis of variance to compare the scores of MCCB among the patients with three different genotypes (CC, TT and CT) of rs3088077 locus. Non-parametric tests were used for those with non-uniform variance. After multiple comparison tests, the results showed that there were no significant differences in MCCB subtests scores among patients with CC, TT and CT genotypes at rs3088077 locus (*P* > 0.05).

Covariance analysis was used to compare the scores of MCCB among the patients with three different genotypes (CC, TT and CT) of rs2303579 locus. The results showed that in the test of WMS-III Spatial Span, the scores of patients with CT genotype were significantly lower than those with CC and TT genotypes (*P* < 0.05). The scores of patients with TT genotype were significantly higher than those with CT genotype (*P* < 0.05) in the test of HVLT-R (Table 5).

**Table 5:** Comparison of MCCB subtest scores in patients with different genotypes of the rs2303579 locus

Subtest	rs2303579			F	*P*	*P*1	*P*2	*P*3
	CC (*n* = 95)	TT (*n* = 67)	CT (*n* = 134)					
TMT-A	48.02 ± 8.599	46.52 ± 18.220	46.09 ± 11.257	0.641	0.527	0.431	0.276	0.889
BACS: Symbol Coding	42.58 ± 10.389	42.82 ± 10.027	40.78 ± 10.012	1.408	0.246	0.850	0.172	0.157
Fluency	49.13 ± 9.905	47.75 ± 12.499	46.64 ± 11.583	1.414	0.245	0.467	0.094	0.470
CPT-IP	42.77 ± 10.616	42.58 ± 9.999	41.12 ± 11.657	0.845	0.431	0.947	0.243	0.333
LNS	46.91 ± 10.070	45.54 ± 10.140	45.22 ± 12.420	0.623	0.537	0.427	0.285	0.911
WMS-III: Spatial Span	45.83 ± 12.232	46.16 ± 11.764	42.46 ± 13.507	2.917	0.056	0.843	0.045*	0.046*
HVLT-R	44.20 ± 11.273	47.39 ± 10.113	43.28 ± 11.388	2.786	0.063	0.081	0.587	0.020*
BVMT-R	43.83 ± 11.793	44.22 ± 12.007	42.49 ± 11.764	0.614	0.542	0.832	0.369	0.328
NAB: Mazes	43.88 ± 9.947	43.93 ± 10.458	41.82 ± 10.115	1.195	0.304	0.946	0.167	0.247
MSCEIT: Managing Emotions	48.33 ± 11.862	48.12 ± 12.957	47.60 ± 11.574	0.078	0.925	0.883	0.695	0.846

Notes: *P*1 value represented the statistical significance between patients with CC genotype and TT genotype. *P*2 value represented the statistical significance between patients with CC genotype and CT genotype. *P*3 value represented the statistical significance between patients with TT genotype and CT genotype. * *P* < 0.05

We used the covariance analysis to compare the scores of MCCB among the three different genotypes of rs11550869 locus, and the same comparison was also performed at rs7162435 and rs62043855 loci. The study of rs62043855 locus showed that patients with TG genotype had significantly lower scores than those with GG genotype (*P* < 0.05) in the test of NAB Mazes, while no significant difference was found between TG and TT genotype in this subtest (*P* > 0.05), neither between TT and GG genotype. In the remaining nine subtests of the MCCB, the scores showed no significant difference between patients with different genotype (TG, TT and GG) of this locus (*P* > 0.05). When it came to rs11550869 and rs7162435 loci, no significant difference between patients with different genotype was found in any subtest of MCCB (*P* > 0.05) (Table 6).

**Table 6:** Comparison of MCCB subtest scores in patients with different genotypes of the rs62043855 locus

Subtest	rs62043855			F	*P*	*P*1	*P*2	*P*3
	TT(*n* = 126)	GG(*n* = 39)	TG(*n* = 131)					
TMT-A	47.30 ± 8.989	47.49 ± 18.530	46.13 ± 13.206	0.706	0.494	0.822	0.243	0.561
BACS: Symbol Coding	42.59 ± 10.301	43.26 ± 9.332	40.66 ± 10.181	2.211	0.111	0.952	0.053	0.161
Fluency	48.60 ± 10.777	47.54 ± 11.480	46.85 ± 11.766	1.014	0.364	0.516	0.158	0.748
CPT-IP	42.23 ± 11.058	42.49 ± 9.965	41.59 ± 11.215	0.400	0.671	0.862	0.379	0.665
LNS	46.83 ± 9.858	45.28 ± 10.105	45.03 ± 12.634	1.962	0.142	0.230	0.058	0.922
WMS-III: Spatial Span	45.20 ± 12.497	45.54 ± 12.553	43.25 ± 13.172	0.949	0.388	0.908	0.215	0.330
HVLT-R	44.41 ± 11.462	47.03 ± 10.786	43.85 ± 10.941	1.246	0.289	0.305	0.453	0.120
BVMT-R	44.14 ± 12.068	45.21 ± 11.225	41.95 ± 11.671	1.669	0.190	0.621	0.144	0.131
NAB: Mazes	43.61 ± 10.299	45.44 ± 9.210	41.60 ± 10.162	2.723	0.067	0.370	0.092	0.03*
MSCEIT: Managing Emotions	47.72 ± 11.473	48.26 ± 14.365	48.08 ± 11.717	0.013	0.987	0.880	0.909	0.942

Notes: *P*1 value represented the statistical significance between patients with TT genotype and GG genotype. *P*2 value represented the statistical significance between patients with TT genotype and TG genotype. *P*3 value represented the statistical significance between patients with GG genotype and TG genotype. * *P* < 0.05

## Discussion

At present, studies have shown that the *NEDD4* gene may be related to the pathological process of schizophrenia. Primary study of our group showed that several loci of the gene may be associated with the occurrence of schizophrenia and the clinical characteristics of patients, however, there is no study to investigate whether the gene is associated with cognitive impairment of patients with schizophrenia. Therefore, we conducted the above studies to analyze whether there is an association between the *NEDD4* gene and cognitive impairment of patients with schizophrenia. The results of the study were analyzed as follows:

The score of WMS-III Spatial Span in MCCB represents the nonverbal working memory of the testee [20]. The multicomponent model of working memory proposed by Baddeley et al. believed that working memory should be elaborated into a three component system, including the visuo-spatial scratchpad, the articulatory loop and the central executive system [21]. At least two types of working memory systems have been discovered, including verbal working memory for processing linguistic information and spatial working memory, which tested by WMS-III Spatial Span in MCCB, for processing spatial information. The spatial working memory plays an important role in the spatial orientation and the solution of visuospatial problems. Glahn found that spatial working memory impairment was one of the neurocognitive deficits that may mark the genetic predisposition to schizophrenia [22], and his findings suggested that deficits of short-term spatial mnemonic processing may be an effective endophenotypic marker for schizophrenia. Combined with our findings, the scores of patients with CT genotype of rs2303579 locus were significantly lower than those with CC and TT genotypes in the test of WMS-III Spatial Span. So it can be considered that patients with CT genotype of rs2303579 locus has more severe spatial working memory impairments, which means the patients with this genotype have more serious defects in temporary storage and initial processing of visual and spatial information than patients with other two genotypes of rs2303579 locus. It is already known that the mutation of C > T at rs2303579 locus can cause amino acid residues to change from Serine to Asparagine [18]. It remains to be further studied whether this amino acid change affects the function of NEDD4 protein, and then leads to the deficiency of spatial working memory in patients with schizophrenia.

The test of HVLT-R in MCCB can be used to test whether verbal memory and learning ability of schizophrenia patients are impaired [20]. Warrick et al. found that people with high-risk schizophrenia showed impaired verbal memory and learning functions before the full expression of psychotic illness, so verbal memory dysfunction should be a genetic endophenotype of the disease [23]. However, Gildas et al. found that depression, low processing speed and selective attention deficits may affect the verbal memory function of patients with schizophrenia, they believed that patient’s verbal memory dysfunction was caused by the above symptoms rather than a primary feature of the disease [24]. Therefore, there is still no unified view on whether the verbal memory dysfunction is the endophenotype of patients with schizophrenia. Combining our analysis results, the scores of patients with TT genotype of rs2303579 locus were significantly higher than those with CT genotype (*P* < 0.05) in the test of HVLT-R. Although there was no significant statistical difference compared with those individuals with CC genotype, the TT genotype individuals also showed an increasing trend (*P* = 0.08), so it can be concluded that patients with TT genotype of rs2303579 locus have better verbal memory than patients with CC and CT genotypes. Therefore, we speculated that rs2303579 locus polymorphism may be associated with verbal memory impairment of patients with schizophrenia. The verbal memory impairment is related to genetic factors and may be one of the endophenotype of this disease.

The cognitive function tested by the test of NAB Mazes in MCCB is the ability of reasoning and problem solving [20]. The ability of reasoning and problem solving is part of the executive functions, which are very important to the appropriate behavior and interact with other cognitive dysfunctions in schizophrenia patients. The NAB Mazes required the patient to retain the information on the pathways that have been previously passed, and then infer the next step. The assessment found that the schizophrenia patient had a defect in the function of the previous information maintaining, which may be one of the important factors for the deficiency of its executive function [25]. The NAB Mazes can be used to reflect the cognitive ability of the prefrontal cortex of the patient, including the patient’s foresight, planning ability, and impulse control ability [26]. The cognitive impairment may be involved with a wide area of the prefrontal cortex, and its specific pathological mechanism has not been elucidated [27]. Combined with our study, patients with TG genotype of rs62043855 locus had a significantly lower score than those with GG genotype (*P* < 0.05) in the NAB. There was no statistically significant difference between TG and TT genotype individuals, but TG genotype individuals also showed a decreasing trend (*P* = 0.092). Therefore, we believed that the genetic polymorphism of rs62043855 locus was associated with the impairment of the ability to reason and solve the problem in schizophrenia patients, the TG genotype patients’ foresight, planning ability, and impulsive control ability were worse than those of other genotypes. As we already know, the mutation of T > G at rs62043855 locus can cause amino acid residues to change from Asparagine to Histidine [18], which may cause changes in the function of NEDD4 protein. However, the relationship among the amino acid residues changing, NEDD4 protein and the impairment of the ability to reason and solve the problem in schizophrenia patients still remains to be further studied.

There were still some limitations in our research. First of all, only the MCCB was used to perform cognitive tests on patients, without other cognitive testing tools such as University of California, San Diego performance-based skill assessment (UPSA), so the cognitive deficits of schizophrenia patients may not be fully reflected. Secondly, our study only analyzed the association between a single locus of the *NEDD4* gene and cognitive dysfunction in schizophrenia. However, schizophrenia is a complex disease with multiple genes involved, therefore, the genetic mechanism of cognitive dysfunction in schizophrenia patients needs to be further discussed in terms of gene-gene interaction and gene-environment interaction.

## Conclusions

In our research, the *NEDD4* gene polymorphism of rs3088077 and rs2303579 loci was associated with schizophrenia in Chinese Han population. The rs2303579 locus was associated with the disorders of spatial working memory and verbal memory in schizophrenia, in which the CT genotype was a risk factor of spatial working memory impairment, and the TT genotype was a protective factor of verbal memory. The rs62043855 locus was associated with the disorders of reasoning skills and problem-solving capacity in schizophrenia, in which the TG genotype was the risk factor of these disorders.

## Data Availability

Researchers interested in the study may contact corresponding author to obtain relevant data via email: liulf521@163.com.

## Abbreviation

AMPA: Α-amino-3-hydroxy-5-methyl-4-isoxazolepropionic acid
BACS: Brief Assessment of Cognition in Schizophrenia
BVMT-R: Brief Visuospatial Memory Test-Revised
CPT-IP: Continuous Performance Test-Identical Pairs
DNA: Deoxyribonucleic acid
DSM-IV: Fourth Edition of the Diagnostic and Statistical Manual for Mental Disorders
E1: Ubiquitin-activating enzyme
E2: Ubiquitin-binding enzyme
E3: Ubiquitin ligase
GluR1: Glutamate receptor 1
HVLT-R: Hopkins Verbal Learning Test-Revised
HWE: Hardy-Weinberg equilibrium
LNS: Letter-Number Span Test
MCCB: Matrics Consensus Cognitive Battery
MSCEIT: Mayer-Salovey-Caruso Emotional Intelligence Test
NAB: Neuropsychological Assessment Battery
NCBI: National Center for Biotechnology Information
*NEDD4*: Neural precursor cell expressed developmentally down-regulated 4
PANSS: Positive and Negative Syndrome Scale
Q-PCR: Quantitative polymerase chin reaction
SCID: Structured Clinical Interview for DSM-IV
SNP: Single-nucleotide polymorphism
TMT-A: Trail Making Test A
Ub: Ubiquitin
UPS: Ubiquitin-proteasome system;
UPSA: University of California, San Diego performance-based skill assessment
WMS-III: Wechsler Memory Scale-Third Edition;
